# A standardized scoring method for the copy of cube test, developed to be suitable for use in psychiatric populations

**DOI:** 10.1186/1744-859X-10-19

**Published:** 2011-07-11

**Authors:** Konstantinos N Fountoulakis, Melina Siamouli, Stamatia Magiria, Panagiotis T Panagiotidis, Sotiris Kantartzis, Vassiliki A Terzoglou, Timucin Oral

**Affiliations:** 1 Third Department of Psychiatry, Aristotle University of Thessaloniki, Thessaloniki, Greece; 2 Asklipios Clinic, Veroia, Greece; 3 School of Medicine, Aristotle University of Thessaloniki, Thessaloniki, Greece; 4 424 General Military Hospital of Thessaloniki, Thessaloniki, Greece; 5 Psychologist, Thessaloniki, Greece; 6 Fifth Inpatient Department of Psychiatry and Outpatient Unit of Mood Disorders, Bakirköy State Teaching and Research Hospital for Neuropsychiatry, Istanbul, Turkey

## Abstract

**Background:**

Although the 'copy of cube test', a version of which is included in the Short Test of Mental Status (STMS), has existed for years, little has been done to standardize it in detail. The aim of the current study was to develop a novel and detailed standardized method of administration and scoring this test.

**Methods:**

The study sample included 93 healthy control subjects (53 women and 40 men) aged 35.87 ± 12.62 and 127 patients suffering from schizophrenia (54 women and 73 men) aged 34.07 ± 9.83 years. The psychometric assessment included the Positive and Negative Symptoms Scale (PANSS) the Young Mania Rating Scale (YMRS), and the Montgomery-Åsberg Depression Rating Scale (MADRS).

**Results:**

A scoring method was developed based on the frequencies of responses of healthy controls. Cronbach's α was equal to 0.75 and inter-rater reliability was 0.90. Three indices and five subscales of the Standardized Copy of the Cube Test (SCCT) were eventually developed. They included the Deficit Index (DcI), which includes the Missing Elements (ME) Mirror Image (M) subscales, the Deformation Index (DfI) which includes the Deformation (D) and the Rotation (R) subscales and the Closing-In Index (CiI).

**Discussion:**

The SCCT seems to be a reliable, valid and sensitive to change instrument for the testing of psychiatric patients. The great advantage of this instrument is the fact that it only requires paper and a pencil, and is this easily administered and brief. Further research is necessary to test its usefulness as a neuropsychological test.

## Background

The copy of cube task is a well known, simple paper and pencil test which is part of the Short Test of Mental Status (STMS) [1,2]. Additionally, patterns of blocks of cubes are incorporated in the Bender Gestalt Test [3-11]. This simple test demands the copy of a Necker cube. This shape is an optical illusion first published in 1832 by the Swiss crystallographer Louis Albert Necker, and it is an ambiguous line drawing. In essence, it is a wireframe drawing of a cube in isometric perspective. This means that parallel edges of the cube are drawn as parallel lines in the picture. The ambiguity lies in the fact that when two lines cross, the picture does not show which is in front and which is behind. This leads to what is called multistable perception, since sometimes the observer might experience the cube 'flipping' between its two perceptual solutions.

This phenomenon is very interesting as it shows that from an ambiguous picture, the human visual system picks an interpretation of each part that makes the whole consistent. Humans do not usually see an inconsistent interpretation of the cube (for example, an impossible object). Most people see the lower-left face as being in front, possibly because people view objects from above, with the top side visible, far more often than from below with the bottom visible, so the brain selects as most probable the interpretation that the cube is viewed from above. Thus, the use of the Necker cube in neuropsychology has shed light on the human visual system. The phenomenon has served as evidence of the human brain being a neural network with two distinct and equally possible interchangeable stable states [12].

**Figure 1:**
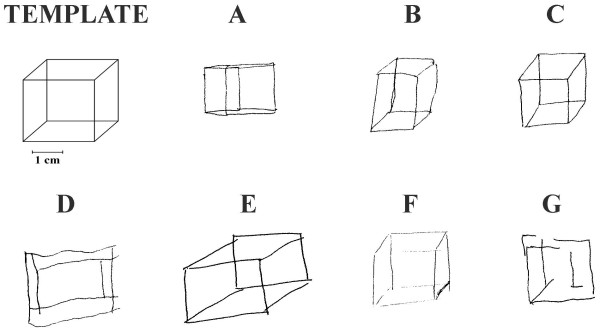
**Samples showing how patients with schizophrenia perform in the Necker cube test**.

The scoring method as indicated in the STMS rates the performance from 0-2. Psychiatric patients, however, including most patients with schizophrenia, are likely to receive a score of 1 or 2, which is largely similar to controls. Samples showing how patients with schizophrenia perform in this task are shown in Figure 1. It is obvious that by using these scoring methods to assess the drawings of psychiatric patients, valuable information might be lost.

The reversal of the perception of the Necker cube has been extensively studied, but this is not the case concerning its copying. To date no standardized method has been developed. The aims of the current study were to develop a novel and detailed standardized method of administration and scoring of the copy of the Necker cube test and to preliminarily test this method in schizophrenic patients. This new scoring method aims to be reliable, valid and sensitive to change in response to treatment.

## Methods

### Study sample

The study sample included 93 healthy control subjects (53 women (56.98%) and 40 men (43.02%)) aged 35.87 ± 12.62 and 127 patients suffering from schizophrenia according to *Diagnostic and Statistical Manual of Mental Disorders *fourth edition, text revision (DSM-IV-TR) criteria (54 women (42.52%) and 73 men (57.48%)) aged 34.07 ± 9.83.

All subjects were physically healthy with normal clinical and laboratory findings. All control subjects and patients gave informed consent and the protocol received approval from the University's Ethics Committee. The patients were either inpatients or outpatients of a private psychiatric clinic.

### Clinical diagnosis

The diagnosis was set according to DSM-IV-TR criteria on the basis of a semistructured interview based on the Schedules for Clinical Assessment in Neuropsychiatry (SCAN) version 2.0 [13].

### The SCCT procedure

The SCCT procedure required the subject copy a Necker cube. The template shape is shown in Figure 1 and in Additional file 1. The SCCT instructions ask the subject to draw an identical shape on the same piece of paper. The template shape was printed on the left half of the sheet leaving space for the subject to reproduce it on the right. No time limit was set and no time recording was made.

The assessment included the Random Letter Test for the assessment of attention and vigilance [14] to assure that subjects could concentrate enough. This includes the following four series' of letters: LTPEAOAISTDALAA, ANIABFSAMPZEOAD, PAKLATSXTOEABAA and ZYFMTSAHEOAAPAT. The first and third group include five 'A's, while the second and the fourth include four 'A's. The test requires the patient to hit the desk when the examiner pronounces 'A'. Errors of omission and commission are recorded. It is expected (and verified in the present study) that the mean number of errors expected from healthy controls in this test is around 0.2. Both errors of omission and commission were registered for this test.

### The psychometric assessment

The psychometric assessment included Positive and Negative Symptoms Scale (PANSS) [15], the Young Mania Rating Scale (YMRS) [16], and the Montgomery-Åsberg Depression Rating Scale (MADRS) [17] in order to assess the clinical picture of patients. The PANSS assesses psychotic symptoms, the YMRS manic symptoms and the MADRS depressive symptoms.

### Raters

All authors served as raters with regard to the psychometric scales and neuropsychological testing. They were not blind to clinical diagnosis. Only brief training was given, as all of them were already experienced in the field. There was no specific training concerning the SCCT because the essence of the development procedure was that the scoring directions included in the test should be sufficient alone.

### Statistical analysis

The statistical analysis included the development of frequency tables for scores of healthy controls so as to arrive at percentile scores and develop a scoring method for the scale. The Pearson's R correlation coefficient, factor analysis (varimax normalized rotation) and item analysis [18] (calculation of Cronbach's α) were used to explore the internal structure of the scale. Analysis of variance [19], was used to test the difference between groups, and was performed separately for subjects below and above the age of 40. Discriminant function analysis was also used to explore differences between groups and the power of the scale in discriminating between them. The Pearson's R correlation coefficient was calculated to assess the inter-rater reliability. However, the calculation of correlation coefficients is not a sufficient method to test reliability and reproducibility of a method and its results, because it is an index of correlation and not an index of agreement [19-21]. The calculation of means and standard deviations for each SCCT item and total score during the rating by each examiner may provide an impression of the stability of results.

Additionally, the means and the standard deviations of the differences concerning each SCCT item between rating and re-rating were calculated and the plots of the rating vs re-rating and difference vs average value for each variable were created. In fact it is not possible to use statistics to define acceptable agreement [19]. However, these plots may assist decision. This method has been used in previous studies concerning the validation of scientific methods [22,23].

## Results

The frequency tables for scores of healthy controls are shown in Table 1. In the same table the proposed scoring for each item is also shown. This scoring method is based on the frequencies of responses of healthy controls (percentile scores).

**Table 1:** Frequencies of healthy controls' performance in each item and proposed standardized score

Raw score	No. of observations	%	Standard score
Number of 'A' omissions			

0	92	98.92	100

1	1	1.08	0

>1	0	0.00	0

Total	93	100.00	

Number of 'A' intrusions			

0	86	92.47	100

1	6	6.45	8

2	1	1.08	1

>2	0	0.00	0

Total	93	100.00	

Missing lines (maximum 12)			

0	90	96.77	100

1	2	2.15	2

2	1	1.08	1

>2	0	0.00	0

Lines which are not parallel			

0	34	36.56	100

1	17	18.28	65

2	20	21.51	45

3	12	12.90	25

4	8	8.60	10

5	2	2.15	2

>5	0	0.00	0

Distorted lines			

0	11	11.83	100

1	15	16.13	90

2	18	19.35	70

3	10	10.75	55

4	15	16.13	40

5	10	10.75	25

6	5	5.38	15

7	4	4.30	10

8	1	1.08	5

9	3	3.23	4

10	1	1.08	1

>10	0	0.00	0

Missing angles (maximum 26)			

0	91	97.85	100

1-10	2	2.15	2

>10	0	0.00	0

Number of right angles which are not (maximum 12)			

0	44	47.31	100

1	16	17.20	50

2	5	5.38	35

3	6	6.45	30

4	3	3.23	25

5	5	5.38	20

6	7	7.53	15

7	1	1.08	7

8	3	3.23	6

9	1	1.08	3

10	1	1.08	2

11	0	0.00	2

>11	1	1.08	1

Angles with different size than the template (maximum 26)			

0	30	32.26	100

1	15	16.13	70

2	9	9.68	50

3	8	8.60	40

4	4	4.30	35

5	5	5.38	30

6	5	5.38	25

7	4	4.30	18

8	4	4.30	15

9	1	1.08	10

10	3	3.23	8

11	1	1.08	5

12	1	1.08	4

13	1	1.08	3

14	0	0.00	3

15	1	1.08	2

16	1	1.08	1

>16	0	0.00	0

Missing elements (maximum 7)			

0	90	96.77	100

1	2	2.15	2

2	0	0.00	2

3	1	1.08	1

>3	0	0.00	0

Distorted elements (maximum 7)			

0	19	20.43	100

1	22	23.66	80

2	13	13.98	55

3	14	15.05	40

4	9	9.68	27

5	8	8.60	17

6	1	1.08	8

7	7	7.53	7

Elements 1 and 2			

0	31	33.33	100

1	62	66.67	67

Elements 3, 4, 5 and 6			

0	13	13.98	100

1	10	10.75	85

2	22	23.66	75

3	48	51.61	50

Three-dimensional level missing			

0	90	96.77	100

1	3	3.23	3

2	0	0.00	0

Rotation			

No	74	79.57	100

Yes	19	20.43	20

Mirror Image			

No	89	95.70	100

Yes	4	4.30	4

Close-In			

No	93	100.00	100

Yes	0	0.00	0

Subjects were divided into those under and over the age of 40 (for those bellow the age of 40: controls 28.57 ± 7.18 years old vs patients 30.18 ± 6.30 years old, *P *= 0.09 and for those above the age of 40: controls 50.70 ± 6.90 years old vs patients 55.60 ± 9.90 years old, *P *= 0.001). The one-way analysis of variance (ANOVA) revealed significant results for subjects under the age of 40 (*P *< 0.001) but not for those above this age (*P *= 0.055). Note that SCCT-14 had no variance so it was not included in the analysis. The results are shown in Table 2 along with post hoc tests. This analysis made the samples considerably smaller and, thus, this study does not have adequate power to detect a difference between healthy controls and people with schizophrenia in those over 40 and testing should be considered exploratory. The results indicate that the difference between healthy controls and patients with schizophrenia gets smaller with age because the performance of controls gets worse, even though patients were significantly older in the above 40 years old group.

**Table 2:** Comparison of the scores of healthy controls and schizophrenic patients above and below 40 years of age, with t test as the post hoc test

	Controls		Patients with Schizophrenia		
	**Mean**	**SD**	**Mean**	**SD**	***P value***

Below 41 years	N = 60		N = 101		

RLT-A	100.00	0.00	71.43	45.72	<0.001

RLT-B	84.14	21.31	65.00	40.05	<0.001

SCCT-1	100.00	0.00	79.16	40.67	<0.001

SCCT-2	63.10	35.20	41.70	35.12	<0.001

SCCT-3	57.06	30.57	41.14	29.21	0.001

SCCT-4	100.00	0.00	71.83	44.76	<0.001

SCCT-5	66.42	36.94	31.45	30.23	<0.001

SCCT-6	60.52	34.46	45.74	38.96	<0.05

SCCT-7	100.00	0.00	76.39	42.44	<0.001

SCCT-8	56.95	31.41	40.10	30.86	<0.001

SCCT-9	77.11	15.34	79.89	16.18	NS

SCCT-10	68.63	19.27	65.43	19.66	NS

SCCT-11	100.00	0.00	79.30	40.40	<0.001

SCCT-12	85.81	30.81	84.76	31.56	NS

SCCT-13	95.35	20.77	85.37	34.67	<0.05

SCCT-14	100.00	0.00	100.00	0.00	NS

SCCT	1,128.77	145.22	922.26	212.89	<0.001

Deficit Index (DcI)	496.63	17.82	395.61	150.78	<0.001

Missing Elements (ME)	400.00	0.00	310.82	153.23	<0.001

Mirror Image (M)	96.63	17.82	84.79	35.23	<0.05

Deformation Index (DfI)	182.60	34.11	169.74	47.19	NS

Deformation (D)	450.14	138.94	343.06	137.32	<0.001

Rotation (R)	85.96	30.70	84.95	31.42	NS

Closing-In Index (CiI)	100.00	0.00	100.00	0.00	NS

Above 40 years	N = 33		N = 26		

RLT-A	96.77	17.96	84.62	37.55	NS

RLT-B	87.13	15.98	62.46	43.00	<0.01

SCCT-1	93.65	24.60	77.23	42.40	NS

SCCT-2	61.68	29.48	39.65	38.03	<0.05

SCCT-3	53.39	30.58	27.69	26.87	<0.01

SCCT-4	93.68	24.47	58.38	49.56	<0.001

SCCT-5	58.00	38.12	33.12	26.16	<0.01

SCCT-6	54.65	35.07	32.15	30.93	<0.05

SCCT-7	93.65	24.60	73.46	44.59	<0.05

SCCT-8	58.61	32.58	31.77	31.08	<0.01

SCCT-9	78.71	16.05	77.15	15.53	NS

SCCT-10	63.23	18.42	56.73	15.10	NS

SCCT-11	90.61	29.15	73.42	44.66	NS

SCCT-12	79.35	35.58	72.31	38.81	NS

SCCT-13	96.90	17.24	96.31	18.83	NS

SCCT-14	100.00	0.00	100.00	0.00	NS

SCCT	1,076.10	190.59	849.38	188.07	<0.001

Deficit Index (DcI)	470.19	93.07	368.60	164.78	<0.01

Missing Elements (ME)	375.53	91.84	271.80	164.47	<0.01

Mirror Image (M)	94.67	22.30	96.80	17.53	NS

Deformation Index (DfI)	174.67	38.89	170.13	46.17	NS

Deformation (D)	427.53	143.18	312.57	126.38	0.001

Rotation (R)	80.00	35.13	73.33	38.36	NS

Closing-In Index (CiI)	100.00	0.00	100.00	0.00	NS

NS = not significant; RLT = random letter test; SCCT = Standardized Copy of the Cube Test.

The Pearson's R correlation coefficients among the SCCT items in the total study sample are shown in Table 3.

**Table 3:** Pearson Correlation coefficients (R) among the Standardized Copy of the Cube Test (SCCT) items and random letter test (RLT) scores in the total study sample

	SCCT-1	SCCT-2	SCCT-3	SCCT-4	SCCT-5	SCCT-6	SCCT-7	SCCT-8	SCCT-9	SCCT-10	SCCT-11	SCCT-12	SCCT-13	SCCT
RLT-A	**0.56**	**0.22**	-0.03	**0.59**	**0.26**	**0.23**	**0.58**	0.10	-0.10	0.02	**0.53**	-0.02	-0.10	**0.45**

RLT-B	**0.37**	**0.23**	0.00	**0.29**	**0.25**	**0.20**	**0.33**	0.13	-0.03	0.09	**0.42**	0.05	0.03	**0.37**

SCCT-1		0.12	0.07	**0.79**	**0.15**	**0.20**	**0.85**	0.05	**-0.23**	**-0.18**	**0.84**	-0.03	-0.08	**0.62**

SCCT-2			**0.37**	**0.20**	**0.55**	**0.45**	**0.16**	**0.57**	**0.21**	**0.38**	**0.13**	0.05	-0.07	**0.62**

SCCT-3				**0.16**	**0.41**	**0.15**	0.10	**0.39**	**0.13**	**0.21**	0.09	0.03	0.05	**0.47**

SCCT-4					**0.21**	**0.19**	**0.86**	0.12	**-0.16**	-0.10	**0.71**	-0.05	-0.12	**0.66**

SCCT-5						**0.39**	**0.18**	**0.52**	**0.18**	**0.25**	**0.21**	**0.16**	0.06	**0.65**

SCCT-6							**0.23**	**0.62**	**0.29**	**0.44**	**0.19**	**0.15**	-0.04	**0.63**

SCCT-7								0.09	**-0.22**	**-0.15**	**0.77**	-0.09	-0.10	**0.65**

SCCT-8									**0.38**	**0.59**	0.08	**0.15**	0.10	**0.67**

SCCT-9										**0.48**	**-0.19**	0.03	-0.05	**0.18**

SCCT-10											-0.10	**0.16**	0.04	**0.36**

SCCT-11												0.00	-0.05	**0.63**

SCCT-12													-0.02	**0.22**

SCCT-13														0.08

Values significant at *P *< 0.05 are marked in bold. The variable SCCT-14 was excluded because of lack of variability.

The Pearson's R correlation coefficient, among the SCCT items and the PANSS (Positive, Negative and General Psychopathology scales), the YMRS and the MADRS are shown in Table 4.

**Table 4:** Pearson Correlation coefficients (R) among the SCCT items and the psychometric scales scores, in schizophrenic patients

	PANSS-Positive	PANSS-Negative	PANSS-General psychopathology	YMRS	MADRS
RLT-A	0.00	0.06	0.08	-0.14	-0.11

RLT-B	-0.02	-0.03	-0.04	0.07	**-0.16**

SCCT-1	-0.12	**-0.16**	-0.15	-0.03	-0.14

SCCT-2	-0.15	**-0.20**	**-0.21**	-0.13	-0.14

SCCT-3	-0.11	**-0.18**	-0.13	0.01	-0.08

SCCT-4	**-0.30**	**-0.34**	**-0.31**	**-0.39**	**-0.18**

SCCT-5	**-0.33**	**-0.29**	**-0.33**	**-0.18**	**-0.25**

SCCT-6	**-0.16**	-0.09	-0.15	-0.01	-0.11

SCCT-7	**-0.17**	**-0.20**	**-0.19**	-0.10	-0.13

SCCT-8	**-0.23**	**-0.17**	**-0.23**	-0.10	-0.14

SCCT-9	0.00	0.05	0.00	0.10	-0.03

SCCT-10	-0.13	-0.13	-0.14	-0.05	-0.10

SCCT-11	-0.14	**-0.20**	**-0.19**	0.00	**-0.23**

SCCT-12	-0.06	-0.01	-0.12	-0.03	**-0.19**

SCCT-13	**-0.18**	**-0.21**	**-0.22**	-0.04	**-0.21**

SCCT-14					

SCCT total	**-0.33**	**-0.34**	**-0.37**	**-0.16**	**-0.30**

Deficit Index (DcI)	**-0.25**	**-0.30**	**-0.28**	**-0.16**	**-0.23**

Missing Elements (ME)	**-0.21**	**-0.25**	**-0.23**	-0.15	**-0.19**

Mirror Image (M)	**-0.18**	**-0.21**	**-0.22**	-0.04	**-0.21**

Deformation Index (DfI)	-0.15	-0.13	**-0.22**	-0.05	**-0.27**

Deformation (D)	**-0.25**	**-0.23**	**-0.27**	-0.10	**-0.19**

Rotation (R)	-0.06	-0.01	-0.12	-0.03	**-0.19**

Closing-In Index (CiI)					

Close-In (CI)	-		-	-	-

Values significant at *P *< 0.05 are marked in bold. Item 14 has no variance so a correlation coefficient cannot be calculated for it.

MADRS = Montgomery-Åsberg Depression Rating Scale; PANSS = Positive and Negative Symptoms Scale; RLT = random letter test; SCCT = Standardized Copy of the Cube Test; YMRS = Young Mania Rating Scale.

The results of the factor analysis (varimax normalized rotation) are shown in Table 5. The analysis (by using the Keiser-Fleish criterion of eigenvalues larger than 1) produced four factors explaining 71% of the total variance. The scores in the subscales created on the basis of these factors and the differences between groups in these scales are also shown in Table 6. The last SCCT item (closing in) was included as a fifth subscale, since it did not contribute to the factor analysis. The one-way ANOVA revealed significant differences between the two diagnostic groups and post hoc tests showed that this difference concerned the some of the subscales but not all (*P *< 0.001; Table 6).

**Table 5:** Factor analysis of Standardized Copy of the Cube Test (SCCT) items (varimax normalized rotation) of the whole sample

	Factor 1	Factor 2	Factor 3	Factor 4
SCCT-1	**0.94**	-0.01	0.03	0.02

SCCT-2	0.17	**0.74**	-0.12	-0.11

SCCT-3	0.10	**0.49**	-0.49	-0.23

SCCT-4	**0.89**	0.10	0.02	-0.07

SCCT-5	0.21	**0.63**	-0.37	0.07

SCCT-6	0.21	**0.71**	0.15	0.20

SCCT-7	**0.94**	0.05	0.03	-0.06

SCCT-8	0.05	**0.85**	-0.15	0.09

SCCT-9	-0.31	**0.60**	0.31	-0.09

SCCT-10	-0.23	**0.73**	0.11	0.13

SCCT-11	**0.89**	0.04	-0.02	0.05

SCCT-12	-0.03	0.13	-0.04	**0.94**

SCCT-13	-0.14	-0.06	**-0.76**	0.08

SCCT-14	-	-	-	-

Proportion total	28%	26%	9%	8%

Total variance explained				71%

**Table 6:** Comparison between the two diagnostic groups (one-way analysis of variance (ANOVA)) concerning SCCT subscales

	Healthy controls		Patients with schizophrenia		
	
	Mean	SD	Mean	SD	*P value*
Deficit Index (DcI)	486.40	60.47	386.87	154.59	<0.001

Missing Elements (ME)	390.53	57.90	298.96	156.95	<0.001

Mirror Image (M)	95.87	19.58	87.91	31.97	<0.05

Deformation Index (DfI)	179.53	36.04	170.13	46.50	NS

Deformation (D)	441.29	140.27	334.48	134.20	<0.001

Rotation (R)	83.66	32.43	82.22	33.38	NS

Closing-In Index (CiI):					

Close-In (CI)	100.00	0.00	100.00	0.00	NS

The correlation coefficients among these subscales are shown in Table 7 and they are non-significant. A second factor analysis of these subscales produced two superfactors explaining 29% and 28% of total variance respectively (Table 8).

**Table 7:** Correlation coefficients among the SCCT subscales

	Mirror Image (M)	Deformation (D)	Rotation (R)
Missing Elements (ME)	-0.10	0.15	-0.05

Mirror Image (M)		0.02	-0.02

Deformation (D)			0.15

Close-In (CI)	-	-	-

**Table 8:** Factor analysis of the subscales (second order factor analysis)

	Second-order factor 1	Second-order factor 2
Deficit Index (DcI)		

Missing Elements (ME)	0.15	**-0.80**

Mirror Image (M)	0.12	**0.61**

Deformation Index (DfI)		

Deformation (D)	**0.76**	-0.24

Rotation (R)	**0.74**	0.24

Closing-In Index (CiI)		

Close-In (CI)	-	-

Explained variance	1.16	1.12

Proportion of variance explained	29%	28%

Total variance explained		57%

Significant values are in bold. Because of lack of variability the CI subscale was not included in the analysis.

Item analysis (calculation of Cronbach's α) Cronbach's α was equal to 0.75, with no item increasing dramatically the α coefficient when omitted.

The discriminant function analysis results are shown in Tables 9 and 10. This analysis produced the following function: when 2 × (SCCT-4) + 3 × (SCCT-5) + 2 × (SCCT-13) = >363.6 then the subject is likely to be a healthy control rather than a schizophrenic patient. This function correctly classified 62.36% of controls and 89.76% of patients with schizophrenia, which is a satisfactory performance.

**Table 9:** Discriminant function analysis results and function coefficients

Diagnosis	Percentage classified correct	Classified as healthy controls	Classified as schizophrenic patients	Total
Healthy controls	62.36	58	35	93

Schizophrenic patients	89.76	13	114	127

Total	78.18	71	149	220

**Table 10:** Discriminant function analysis results and function coefficients

	Healthy control function coefficients	Schizophrenic patient function coefficients
Constant	-40.8311	-37.1956

SCCT-4	0.0034	-0.0189

SCCT-5	0.0058	-0.0194

SCCT-13	0.1766	0.1615

SCCT = Standardized Copy of the Cube Test.

The Pearson's R correlation coefficient (R) for inter-rater reliability is 0.90 for the total SCCT scale and ranges from 0.51 to 0.90 for individual items (Table 11). The calculation of means and standard deviations for each SCCT item and total score for the rating and re-rating as well as the respective plots and plots of difference vs average value for each variable suggested that the SCCT is reliable.

**Table 11:** Inter-rater reliability coefficients

Item	Inter-rater reliability (N = 35)
SCCT-1	0.90

SCCT-2	0.66

SCCT-3	0.78

SCCT-4	0.87

SCCT-5	0.73

SCCT-6	0.51

SCCT-7	0.82

SCCT-8	0.76

SCCT-9	0.78

SCCT-10	0.76

SCCT-11	0.87

SCCT-12	0.72

SCCT-13	0.86

SCCT-14	-

SCCT total	0.90

Deficit Index (DcI)	0.93

Missing Elements (ME)	0.93

Mirror Image (M)	0.86

Deformation Index (DfI)	0.66

Deformation (D)	0.83

Rotation (R)	0.72

Closing-In Index (CiI)	-

Close-In (CI)	-

CiI has no variability.

SCCT = Standardized Copy of the Cube Test.

## Discussion

The SCCT is a test of visual-motor ability and, although several decades have passed since the copy of a cube test was introduced, little has been done to standardize it. This may be due to the complex pattern of these tests and a preference of the examiners to score them on the basis of an 'overall' impression or 'qualitatively'. Little data can be found in the literature and even then only because it is included in the STMS [1,2]. The Bender Gestalt Test includes complex three-dimensional figures constituted from many Necker cubes, but again scoring is simplistic [3-5,8-11]. Scoring is based on the overall impression and quality of the drawing as well as on common errors observed, and the focus is on detecting 'organic' brain defects. However, in this way many details in the performance of patients may be lost, and this is especially true when the test is used in psychiatric populations.

**Figure 2:**
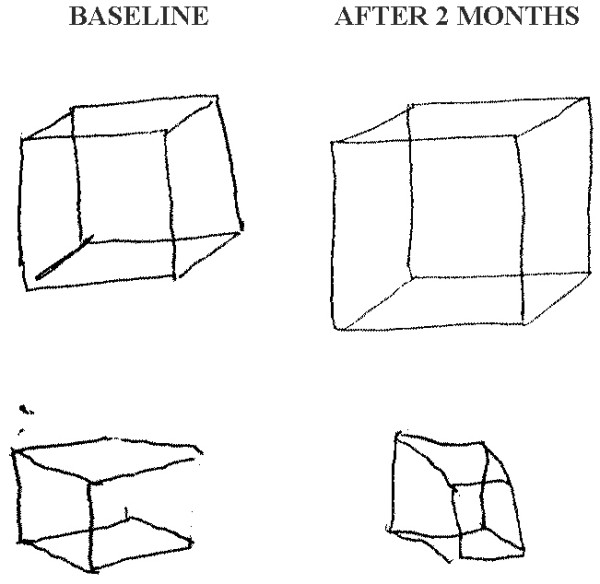
**Examples of how performance on the Standardized Copy of the Cube Test (SCCT) changes after 2 months of antipsychotic treatment**.

The current study attempted to develop a standardized scoring method that would allow the examiner to reliably quantify the subject's performance in the copy the Necker cube test. This test requires the subject to copy a simple drawing template. Both the drawing template and the resulting SCCT along with the scoring method developed by the current study are shown in Additional file 1. The test and its scoring method proved to be reliable and stable. There are some clues that it could be also sensitive to change after treatment. An example of possible change after 2 months of antipsychotic treatment is shown in Figure 2. However, targeted research is necessary to show whether this is the case and also it is necessary to apply the test to different patient population, especially to patients suffering from 'organic' brain disease, before and after therapeutic intervention.

The scoring method is such that allows for maximum contrast and differentiation between healthy subjects and patients and simultaneously leaves little space for subjective assessment. Largely, the scoring method expands levels 2-4 of the Bender-Gestalt scoring system. Further research is necessary to show whether such a detailed approach adds substantially to the understanding of the neurocognitive deficit of mental patients or simply consumes time.

The results of the discriminant function analysis support the usefulness of this new scoring method. By using the functions, the SCCT can assist in the differentiation between patients with schizophrenia from healthy controls. However, apart from discriminant function analysis, we did not proceed to try to calculate sensitivity and specificity for one or more specific cut-off points, because the overlap between groups was significant and the test seems to be useful to assess aspects of cognitive function but not as a specific diagnostic test for a specific illness.

The correlation coefficients among individual SCCT items, although some were significant, suggest that overall each item assesses a distinct issue. This is also reflected in factor analysis. The four factors that emerge explain 71% of the total variance. The SCCT can be divided into subscales on the basis of the factor analysis and its interpretation. In this way, five subscales can be created. The first factor includes items 1, 4, 7 and 11 and it constitutes the Missing Elements (ME) subscale. The second includes items 2, 3, 5, 6, 8, 9 and 10 and it constitutes the Deformation (D) subscale. The third includes only item 13 and it constitutes the Mirror (M) subscale. The fourth includes only item 12 and constitutes the Rotation (R) subscale. Item 14 had no variability and thus it constitutes a separate subscale, the Close-In (CI) subscale.

Correlations among these subscales are very weak. The factor analysis of these subscales produced three superfactors, named 'indices'. The first (subscales ME and M) constitutes the 'Deficit Index' (DcI), while the second (subscales D and R) is the 'Deformation Index' (DfI). The third index (subscale CI alone) is the 'Closing-In Index' (CiI). It is important to note that all the items of the SGST included in the DcI are easy for the healthy subject, while the more difficult ones (2, 5 and 8) are included in the DfI. Patients differ from controls concerning DfI and CiI indices (*P *< 0.001) but not DcI. In the frame of the above, the SCCT is divided into the following three indices and five subscales:

(a) Deficit Index (DcI), which includes the following two subscales: (1) Missing Elements (ME) subscale (items 1, 4, 7 and 11); (2) Mirror Image (M) subscale (item 13).

(b) Deformation Index (DfI), which includes the following two subscales: (3) Deformation (D) subscale (items 2, 3, 5, 6, 8, 9 and 10)); (4) Rotation (R) subscale (item 12).

(R) Closing-In Index (CiI), which includes the following subscale: (5) Close-In (CI) subscale (item 14).

Further research is necessary to elucidate the underlying cognitive functions and deficits that are reflected in these indices and subscales. The correlations among the psychometric scales (PANSS, YMRS and the MADRS) and individual items and subscales of the SCCT revealed some very interesting points (Table 4). The Deficit Index correlates negatively with all psychometric scales. The MADRS correlates also negatively with all subscales and indices. Generally the correlation among the scoring of the SCCT and the psychometric scales is significant. The above suggest a complex neurocognitive profile for schizophrenia as this is revealed by the SCCT. Further research is necessary to uncover specific issues and mechanisms. Commenting on these correlations is beyond the scope of the current manuscript and the data included here are insufficient as they do not focus on this research target.

We believe that further factor analysis with the inclusion of different patient groups will help to further elucidate the mechanisms underlying performance in the SCCT.

## Conclusions

The current study has developed a reliable, valid and maybe sensitive to change instrument. The great advantage of this instrument is the fact that it only requires paper and a pencil, and hence is easily administered and brief. Further research is necessary to test its usefulness as a neuropsychological test.

## Competing interests

The authors declare that they have no competing interests.

## Authors' contributions

KNF designed the study, analyzed the data, interpreted the results, wrote the draft and subsequent versions and finalized the manuscript. MS collected data, assisted in the interpretation of results, gave input to revisions of the manuscript and approved the final version. PTP collected data, assisted in the interpretation of results, gave input to revisions of the manuscript and approved the final version. StM collected data, assisted in the interpretation of results, gave input to revisions of the manuscript and approved the final version. SK collected data, assisted in the interpretation of results, gave input to revisions of the manuscript and approved the final version. VAT collected data, assisted in the interpretation of results, gave input to revisions of the manuscript and approved the final version. TO collected data, assisted in the interpretation of results, gave input to revisions of the manuscript and approved the final version.

## Supplementary Material

Additional file 1 **Standardized Copy of the Cube Test (SCCT)**. The SCCT. Click here for file
