# Supplementary material for: A standardized scoring method for the copy of cube test, developed to be suitable for use in psychiatric populations
Source: Ann Gen Psychiatry. 2011 Jul 11;10:19. doi: 10.1186/1744-859X-10-19 (PMC3154184; doi:10.1186/1744-859X-10-19)
Supplement: Additional file 1 — Standardized Copy of the Cube Test (SCCT). The SCCT. [file 1744-859X-10-19-S1.DOC]

**Additional file 1**

**Standardized Copy of the Cube Test (SCCT)**

***Fountoulakis et al. 2008***

| **Template:** | ***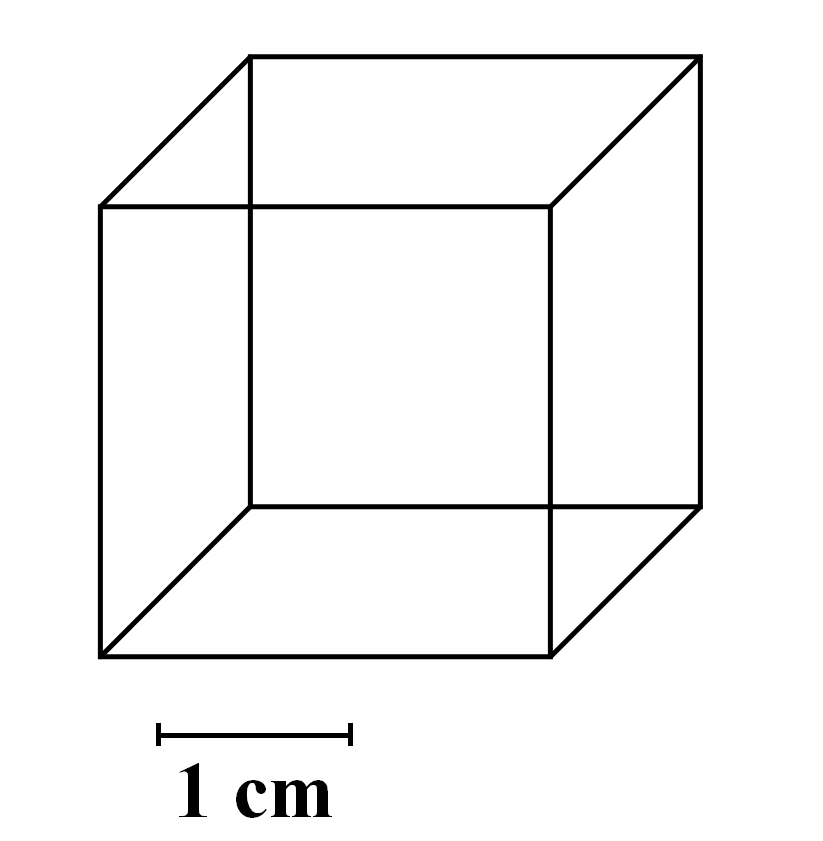*** |
| --- | --- |

# Instruction:

# Please copy the above drawing making a perfect identical one

#

| 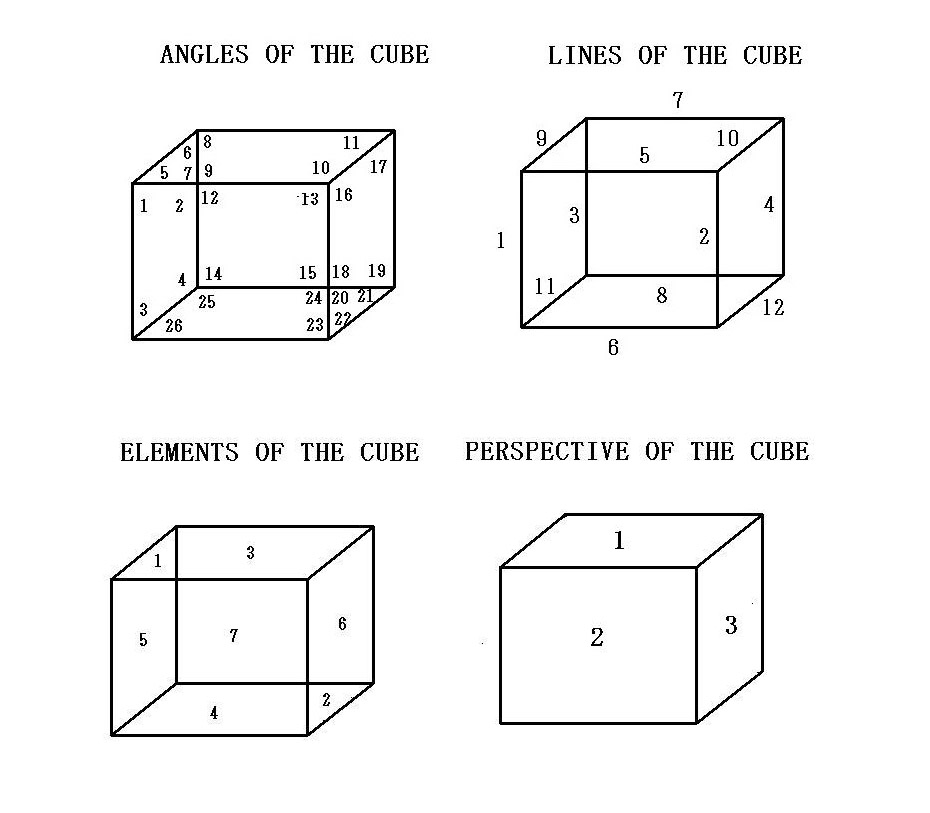 | Total number of lines: 12Total number of angles: 26Total number of right angles: 14(#1, 2, 7, 8, 9, 23, 24, 12, 13, 14, 15, 18, 19, 20)Total number of elements: 7Number of surfaces: 3 |
| --- | --- |

|  |  | Rawscore | Standardized Score |
| --- | --- | --- | --- |
|  | Count missing lines. If the line exists but it is distorted or at a wrong position or with wrong orientation, rate it as present while its deficits will be scored in the following items. (max 12) | 0  1  2  >2 | 100  2  1  0 |
|  | Count the lines which are not parallel to those they should be. | 0  1  2  3  4  5  >5 | 100  65  45  25  10  2  0 |
|  | Count the lines which are distorted (curveted, fragmented as well as corrected). (max 12) | 0  1  2  3  4  5  6  7  8  9  10  >10 | 100  90  70  55  40  25  15  10  5  4  1  0 |
|  | Count missing angles (max 26) | 0  1-10  >10 | 100  2  0 |
|  | Count angles which should be right (90 degrees) and are not. (max 14) | 0  1  2  3  4  5  6  7  8  9  10  11  >11 | 100  50  35  30  25  20  15  7  6  3  2  2  1 |
|  | Count the angles which are significantly different in size in comparison to the template. (max 26) | 0  1  2  3  4  5  6  7  8  9  10  11  12  13  14  15  16  >16 | 100  70  50  40  35  30  25  18  15  10  8  5  4  3  3  2  1  0 |
|  | Count missing elements. (max 7) | 0  1  2  3  >3 | 100  2  2  1  0 |
|  | Count the elements of the cube which do not have the right shape. (max 7) | 0  1  2  3  4  5  6  7 | 100  80  55  40  27  17  8  7 |
|  | The elements # 1 and 2 are similar and should be identical after rotation or mirror transformation. If they are analogous in the drawing cite ‘yes’. If not cite ‘no’. If either or both are missing cite ‘no’ also. | 0  1 | 100  67 |
|  | The elements # 3, 4, 5 and 6 are similar and should be identical after rotation or mirror transformation If they are analogous in the drawing cite ‘0’. If not cite ‘no’. If not then give a raw score equal to the number of different shapes minus 1. If one or more is missing, score only the remaining ones. If there is only one preserved in the drawing then give a score of ‘0’. (max=3) | 0  1  2  3 | 100  85  75  50 |
|  | Give one point for each level of the 3-D perception missing, no matter of the orientation of the cube (The levels are defined in different dimensions and in order for a level to exist it should give the impression of a 3-D perspective). (max=2) | 0  1  2 | 100  3  0 |
|  | The drawing is rotated | No  Yes | 100  20 |
|  | The drawing is a mirror image of the template | No  Yes | 100  4 |
|  | The drawing is closing-in to the template. | No  Yes | 100  0 |
